# Effectiveness of a Mindfulness-Based Professional Development Program for Primary School Teachers in the Czech Republic: A Quasi-Experimental Study

**DOI:** 10.3390/ijerph21121669

**Published:** 2024-12-14

**Authors:** Kamila Dvořáková, Laura García Valladares, Bethany Butzer, Calvin Lange, Mark Greenberg

**Affiliations:** 1 Edna Bennett Pierce Prevention Research Center, Pennsylvania State University, 314 Biobehavioral Health Building, University Park, PA 16802, USA; mxg47@psu.edu; 2 Área de Educación y Formación del Profesorado, Universidad Europea del Atlántico, 39011 Santander, Spain; laura.garcia@uneatlantico.es; 3 School of Psychology, The Alef Trust, Wirral CH63 0HJ, UK; bethany.butzer@aleftrust.org; 4 Department of Psychology, Ludwig-Maximilians Universität München, 80539 München, Germany; calvin.lange@campus.lmu.de

**Keywords:** mindfulness, compassion, stress management techniques, primary school teachers, well-being

## Abstract

Background: Research has shown that 20% of Czech teachers suffer with burnout symptoms and 65% are at risk of burning out, which suggests that it is essential to continue addressing the issue of stress in Czech teachers. The main objective of this study was to evaluate a self-compassion and mindfulness-based professional development program for primary school teachers in the Czech Republic. Methods: Five schools were recruited, two as intervention schools (*n* of teachers = 47) and three as controls (*n* of teachers = 57). Teachers completed questionnaires at three time points: pre-test in September 2018, post-test in November 2018, and a follow-up in April 2019. Results: The results at post-test indicated that teachers in the intervention group scored significantly higher (*p* < 0.05) in self-efficacy and self-compassion, and significantly lower in depression, anxiety and emotional exhaustion, compared to the controls. The intervention teachers were marginally lower (*p* < 0.10) in perceived stress and marginally higher in subjective well-being, compared to the controls. At follow-up, teachers’ subjective well-being in the control group significantly worsened compared to the baseline. However, the intervention group did not show significant changes over time, which suggests a “protective effect” on the intervention group against worsening during the school year. Conclusions: The study suggests that providing teachers with self-compassion and mindfulness practices can lead to beneficial effects on several outcome variables. Further studies need to investigate if these benefits can be sustained and if they affect teachers’ physical health, their relationships with students, and the students’ outcomes.

## 1. Introduction

While some research has shown that 20% of Czech teachers suffer with burnout symptoms and 65% are at risk of burning out [1], a growing body of research, in recent years, has highlighted the potential benefits of integrating mindfulness-related programs into education, particularly for teachers [2,3]. These kinds of programs attempt to equip educators with effective stress management techniques, self-compassion, and greater self-awareness, which can prove vital for their well-being and subsequently enhance the overall school climate and student-teacher relationships [4,5,6]. As a result, mindfulness programs in educational contexts are experiencing increased momentum, as education systems worldwide face mounting challenges and increasing demands. Research in numerous countries shows that teachers report one of the highest levels of occupational stress and burnout on the job and this work-related stress is linked to negative psychological issues, low job satisfaction, absenteeism, and the intention to quit [7]. Research also suggests that teachers who cultivate self-compassion and mindfulness enhance their well-being and work engagement (i.e., they report being more engaged and having a more fulfilling work experience) [8].

Teachers hold a very unique and indispensable role in society, namely the shaping of future generations through the nurturing of young minds. Despite this profound endeavor, only around 31% of teachers feel as if their profession is valued by society, according to the Organization for Economic Co-operation and Development (OECD) average, and the number drops to a staggering 16% among Czech teachers [9]. However, while some teachers may not feel valued by society, the impact of teacher well-being on student outcomes is important and must not be understated [6]. Improving teachers’ well-being and emotional states have profound downstream effects on their students, which, in turn, correlate with increases in academic self-perceptions and increased perceptions of teacher support and involvement amongst students [10,11].

Consequently, much research has been conducted on the effectiveness of teacher well-being interventions in schools, particularly through the use of mindfulness training [12]. Several mindfulness programs have gained popularity and have been implemented in educational settings in recent years, including the Community Approach to Learning Mindfully program (CALM) [2], Stress Management And Resiliency Training in education (SMART) [13], and Cultivating Awareness and Resilience in Education (CARE) [14,15] or Mindful Self-Compassion (MSC) [16]. CARE and MSC are the focus of the current study. The CARE program is a mindfulness-based professional development program for teachers. The CARE program places emphasis on cultivating emotional awareness, reducing stress, and enhancing general teacher well-being in order to improve the learning environment [5]. Additionally, the benefits provided by mindfulness programs extend beyond just teachers to students and other professionals within the school system. Studies have demonstrated that students who have been exposed to mindfulness practices demonstrate increased emotional regulation, reduced anxiety, and enhanced focus [17]. The theoretical model of a prosocial classroom emphasizes the importance of social and emotional competence among teachers, their well-being and development, as well as the development of supportive teacher-student relations [15]. When teachers participate in mindfulness training, they become better equipped to manage the everyday stressors of their jobs, which translates to increased rates of job satisfaction and a reduction in burnout rates [3,18].

The CARE program aims to facilitate this process through the combination of emotional skill instruction, mindful awareness practices, and compassion-building activities, such as guided reflections aimed at generating feelings of care for oneself and others, and mindful listening exercises designed to increase awareness of one’s emotional reactions without acting upon them [15]. The CARE program is a 30 h intensive program conducted over four day-sessions over 4–6 weeks, with the addition of an over-the-phone coaching booster conducted 2 months later [15]. Throughout these 4–6 weeks, teachers are introduced to emotion skill instructions which utilize a combination of didactic instructions and experiential activities, such as reflective practices and role-play scenarios, with the intent of developing teachers’ recognition and awareness of emotional states and their ability to subsequently analyze their emotional patterns [15]. Such practices are intended to help teachers become more aware of the emotional climate in a classroom, become more emotionally self-aware, and become less emotionally reactive when facing challenging students. Additionally, mindfulness practices are also implemented; this consists of deliberate attention training to focus on one’s awareness of the present moment, whilst sustaining an orientation of openness, curiosity, and attempted unbiased acceptance towards experiences [2]. Mindfulness techniques are utilized with the goals of reducing stress [2] and improving psychological well-being among teachers [17]; this allows for greater emotional patience and a greater capacity for providing support to students, as well as increased cognitive performance in teachers, which is proposed to catalyze positive change in the classroom environment [9]. The final cornerstone of the CARE program is to promote empathy and compassion through compassion practices such as “caring practice” and “mindful listening”. “Caring practice” aims to instill sentiments of care for oneself and others by fostering well-being and peace through mental offerings [15]. Mindful listening consists of promoting the ability to listen to another person without judgment, through the practice of purely noticing emotional reactions one might have, such as the urge to interrupt, offer advice, or judge, without acting upon them [15]. Interventions such as the CARE program have shown promise for enhancing teachers’ emotional regulation, mindfulness, and self-compassion, as well as reducing their psychological stress and time urgency [19].

MSC is the other program relevant for this study. MSC is a program mainly focused on enhancing self-compassion. The principal goal is to provide participants with a variety of tools to increase self-compassion in their daily life, as well as to develop general skills of loving-kindness and mindfulness practices [20]. Taking into account the definitions of compassion from several authors, compassion can be defined as a cognitive, affective, and behavioral process characterized by five elements: recognizing suffering; understanding the universality of suffering in human experience; feeling empathy for the person suffering and connecting with the distress; tolerating uncomfortable feelings aroused in response to the suffering person; and the motivation to act to alleviate suffering [21]. Focusing directly on self-care, self-compassion is a process that involves being open to one’s own suffering (without avoiding or disconnecting from it), generating the desire to alleviate it, and facing it in such a way that involves three aspects: kindness and understanding towards oneself, connection with humanity, and mindfulness (as a state of awareness of our thoughts and feelings instead of over-identifying with them) [22]. Research suggests that self-compassion has a strong correlation with well-being, in that self-compassion practice is linked to an increase in positive mind-states like life satisfaction, happiness, connectedness, self-confidence, optimism, curiosity, and gratitude [23].

The MSC is an 8-week program with 2 or 2 1/2 h meetings once a week, and a half-day meditation retreat. The MSC program consists of formal and informal self-compassion practices, experiential exercises, and discussion periods, as well as homework assignments to help participants to be kinder with themselves [20]. The MSC curriculum is composed of sessions that focus on topics such as understanding and experiencing the self-compassion concept, learning to experience difficult emotions, and embracing joy and gratitude. The MSC is structured to move participants from a conceptual understanding of mindfulness and self-compassion to experiencing these concepts for oneself and bringing them into a good daily practice [16]. Interventions such as the MSC program have shown that being mindfully self-compassionate enhanced teachers’ self-awareness and helped the teachers to deal with challenging moments with self-kindness, all of which contributed to their well-being [24]. Being mindfully self-compassionate also allowed teachers to engage in intentional caring practices that fostered relationships and supported the well-being of children and their colleagues; in other words, self-compassion supports teachers in their caring role [25]. Given the enormous demands of the teaching profession, it is important to note that self-compassion can mitigate the effects of teachers’ burnout and empathy fatigue (which is a major cause of burnout for professional caregivers) [26]. Indeed, research has found a relationship between schoolteachers’ self-compassion and school performance in terms of enrollment rate, promotion rate, retention rate, and cohort survival rate [27]. In addition, in that same study, an inverse correlation was found between schoolteachers’ self-compassion and the drop-out rate, school-leavers rate, and repetition rate. Higher levels of self-compassion are also associated with greater teacher resilience [28], as well as with less stressful perceptions of their experience and better sleep quality [29].

In recent years, the benefits for teachers of the use of compassion and mindfulness- based interventions has started to become apparent. Self-compassion is also being demonstrated to increase the well-being of students [30,31]. For example, teachers in Portugal reported a significant increase in mindfulness and emotional regulation competencies, self-efficacy, and well-being and a decrease in burnout symptoms after participating in a mindfulness-based program designed around three principal components: mindfulness practices, emotional self-regulation, and caring practices [32]. In the same study, a significant improvement was found in teachers’ classroom behaviors related to students’ engagement, as well as significant improvements in the students’ perceptions of the quality of their teachers’ involvement in classroom relationships, the self-reported affect, and the social competencies perceived by their parents.

By prioritizing the mental and emotional health of primary school teachers, it is possible to facilitate a far more nurturing and supportive classroom environment aimed at fostering positive teacher-student relationships, thus enriching the learning experience for young people. Furthermore, there is a pressing need to expand contemplative intervention-research efforts to include non-English speaking countries, allowing for a broader understanding of the potential benefits of mindfulness approaches worldwide, making this one of the aims of the current study.

**The current study**. The main objective of this study was to evaluate the effectiveness of a self-compassion and mindfulness-based professional development program for primary school teachers in the Czech Republic. The program was adapted for this study, utilizing components of the CARE and the MSC programs. We hypothesized that teachers who participated in the program would show increases in their well-being, self-efficacy, and self-compassion, as well as reduced levels of stress, depression, anxiety and burnout, compared with teachers who did not participate in the program. We also expected that the benefits for the teachers who participated in the program would be maintained after a 6-month follow-up period.

## 2. Materials and Methods

### 2.1. Participants

The participants in this study were 104 primary school teachers from five public primary schools in the Czech Republic. Recruitment took place in the fall of 2017 through a collaboration between Charles University, the National Institute of Mental Health in the Czech Republic, and the Czech educational system. The intervention schools were recruited first, as these two schools were the first to express a willingness to participate in the intervention. The three control schools were then recruited, with the aim of matching all five schools as closely as possible regarding the size, location, and student population. There were 47 teachers from the two intervention schools and 57 teachers from the three control schools. The average teacher age was 46.53 years, and their average number of years working in education was 18.69. Seventy-seven teachers identified as female, eight as male, and the rest of the participants did not specify their gender. None of the participants had previous experience with mindfulness practices.

### 2.2. Procedure

#### 2.2.1. Intervention Procedures

For this study, two well-known mindfulness programs were adapted to the Czech educational environment: Cultivating Awareness and Resilience in Education (CARE) and Mindful Self-Compassion (MSC). By reviewing, in a thorough manner, the manuals of both programs, the key elements of the content and practices were selected for use in the school environment. Three Czech mindfulness facilitators taught the program as a professional development (PD) program to the intervention teachers. The PD was structured as a two-day program (10 h at the beginning of September 2018), two follow-up classes (2 classes × 2 h at the end of September and October 2018), and a booster (3 h in February 2019), for a total of 17 h of instruction.

The adapted content of the first day of the program included: an introduction to the course and teachers’ expectations; an introduction to emotion recognition; an introduction to mindfulness through mindfulness of the body; learning about stress and its effects; an introduction to mindful listening; and learning about intention setting. The practices of the first day were as follows: feet on the ground; mindfulness of the body; mindful listening; and mindful breathing. The content of the second day included an introduction to self-compassion practices in daily situations and learning about emotions, self-compassion, and their effects. The second day practices were as follows: the intention for the day; mindful stretching; recognising emotions in the body; and gratitude practice. The content of the first follow-up class was as follows: learning about compassionate communication; recognizing and distinguishing emotions and needs; bringing self-compassion into emotion recognition; and learning about self-criticism and how to work with it. The practices of the first follow-up class were as follows: mindful walking; compassionate breathing; and befriending ourselves. The content of the second follow-up class was learning about self-care and self-compassion in the school environment, and the practices consisted of revisiting previous practices. The content of the last part of the adapted program, the booster, included a review of the practices and how to use them in daily life and the creation of a support system. In this last part of the program, the practice element consisted of revisiting previous practices.

In summary, the overall curriculum focused on the following: (1) psychoeducation about stress and its effects on teachers’ health, the school climate, and their students; (2) stress management benefits and specific self-compassion, emotion regulation, and mindfulness techniques that can be used during the school day; and (3) deepening teachers’ abilities to use these practices sustainably.

#### 2.2.2. Data Collection Procedures

Participants completed the self-report questionnaires below at three time points: the pre-test in September 2018 (Time 1), the post-test in November 2018 (Time 2), and at the follow-up in April 2019 (Time 3).

### 2.3. Measures

**Teacher well-being**. The Teacher Subjective Wellbeing Questionnaire (TSWQ) [33] is an 8-item self-report instrument for assessing teachers’ well-being. This scale is composed of two subscales: teaching efficacy and school connectedness. A Likert scale is used from 1 (almost never) to 4 (almost always). Previous research has found that the Cronbach alpha internal consistency coefficient was 0.82 for the whole scale [33]. In the current study, the McDonald omega statistic was 0.78 for the whole scale.

**Teacher self-efficacy**. The Teacher Self-Efficacy scale (USE) [34] is a 45-item scale that is used to assess the degree of self-efficacy in teachers in a manner which is considerate of the cultural and historical contexts experienced by Czech teachers. A Likert scale is used from 1 (never) to 5 (always). The USE has demonstrated strong whole-scale reliability in previous research (α = 0.95), along with comparably high reliability for its subscales [34]. For this research, this questionnaire was shortened to 9 items that were chosen in collaboration with the questionnaire’s author. Those items had the highest factor loadings. In the current study, the McDonald omega statistic was 0.81 for the whole scale.

**Self-compassion**. The Czech version of the Self Compassion Scale (SCS-CZ) [35] is a 20-item scale which is used to assess self-compassion in teachers with a consideration for Czech language and cultural influences. The scale includes items measuring self-kindness, self-judgment, common humanity, isolation, mindfulness, and over-identification. A Likert scale is used from 1 (almost never) to 5 (very often). Previous research has found that the SCS-CZ has a Cronbach’s alpha coefficient of 0.89 as well as comparable coefficients for the five subscales [35]. In the current study, the McDonald omega statistic was 0.82 for the whole scale.

**Stress**. The Perceived Stress Scale (PSS) [36] is a 4-item scale that includes questions such as, “In the last month, how often have you felt that you were unable to control the important things in your life?”. A Likert scale is used from 0 (never) to 4 (very often). Previous research has found a Cronbach’s alpha coefficient of 0.85 for the PSS [36]. In the current study, the McDonald omega statistic was 0.80 for the whole scale.

**Depression**. The Patient Health Questionnaire (PHQ-8) [37] is a self-administered questionnaire that assesses symptoms of depression with 8 items that are rated on a Likert scale from 0 (not at all) to 3 (nearly every day). Previous research has found that the PHQ-8 has a high level of sensitivity for depression and a Cronbach’s alpha coefficient of 0.89 [37]. In the current study, the McDonald omega statistic was 0.84 for the whole scale.

**Anxiety**. The Generalized Anxiety Disorder Scale (GAD) [38] is a 7-ítem instrument that is used to assess the subjective experience of anxiety and related symptoms. The questionnaire is scored on a Likert scale from 0 (not at all) to 3 (almost every day). The GAD has shown to have a high level of internal consistency in previous research (α = 0.91) [38]. In the current study, the McDonald omega statistic was 0.87 for the whole scale.

**Teacher burnout**. The Maslach Burnout Inventory (MBI) [39] is a 22-item instrument that is used to assess teachers’ subjective experience of work-related burnout. The scale requests participants to rate their perceived attitudes on a 7-point scale ranging from 0 (never) to 6 (every day). Previous research has found that the MBI demonstrates reliable subscale coefficients (emotional exhaustion: α = 0.91; depersonalization: α = 0.79; personal accomplishment: α = 0.79) [40]. For this study, the subscales used were emotional exhaustion (9 items) and personal accomplishment (8 items). In the current study, the McDonald omega statistic was 0.89 for emotional exhaustion and 0.81 for personal accomplishment.

### 2.4. Data Analysis

The data was analyzed through use of the Jamovi statistical software program, Version 2.3.21 (The jamovi project) [41]. First, a preliminary analysis (using independent-samples *t*-tests) was conducted to examine potential baseline differences between the intervention and the control group on all of the outcome measures. There were no significant differences between the intervention and the control groups on any of the baseline measures.

The primary analysis Involved using analysis of covariance (ANCOVA) to examine post-intervention differences between the intervention and the control groups while controlling for baseline scores. In this analysis, the independent variable was the group (intervention vs. control), the dependent variable was the post-intervention score on the questionnaires, and the covariate was the pre-intervention score on the questionnaires.

A secondary analysis involved using analysis of variance (ANOVA) to explore the trajectory of change across all three time points of data collection. Specifically, mixed-ANOVAs were conducted with one between-subjects factor (group: intervention vs. control) and one within-subjects factor (time: pre-intervention, post-intervention, and follow-up). The dependent variable was the score on the questionnaires at each time point. The main result of interest in these analyses was the interaction between group and time. Significant interactions were probed using post-hoc repeated-measures ANOVAs separately for each group (intervention/control) in order to ascertain which time point(s) differed significantly from one another. An alpha value of *p* < 0.05 was used to denote statistical significance. Due to the pilot nature of the present study, as well as recent calls from statisticians for a more nuanced consideration of *p* values [42], alpha values of *p* < 0.10 were denoted as marginally significant. Partial eta squared (η^2^) was used as a measure of the effect size (where 0.02 indicates a small effect, 0.13 indicates a medium effect, and 0.26 indicates a large effect [43].

## 3. Results

The pre-test, post-test, and follow-up means and standard deviations for all outcome variables for the intervention group and the control group are displayed in Table 1.

### 3.1. Intervention Effects on Well-Being, Self-Efficacy, and Self-Compassion at the Post-Test Time Point

At the post-test time point, participants in the intervention group demonstrated significantly higher scores on the Teacher Self-Efficacy scale (USE) (*F*(1, 73) = 5.36, *p* = 0.02, η^2^ = 0.04) and on the Self Compassion Scale (SCS-CZ) (*F*(1, 72) = 5.53, *p* = 0.02, η^2^ = 0.04). There was a marginally significant effect on the Teacher Subjective Wellbeing questionnaire (TSW), with the intervention teachers reporting higher scores than the control participants (*F*(1, 73) = 2.94, *p* = 0.09, η^2^ = 0.03). Participants in the intervention group also demonstrated marginally higher scores than the controls on the TSW Connectedness subscale (*F*(1, 73) = 3.19, *p* = 0.08, η^2^ = 0.03).

### 3.2. Intervention Effects on Stress, Depression, Anxiety, and Burnout at the Post-Test Time Point

Participants in the intervention group demonstrated significantly lower post-test depression-symptom scores on the Patient Health Questionnaire (PHQ-9) compared to the control group (*F*(1, 73) = 6.01, *p* = 0.02, η^2^ = 0.06). The intervention group also demonstrated significantly lower post-test anxiety-symptom scores on the Generalized Anxiety Disorder Scale (GAD) compared with control participants (*F*(1, 73) = 10.42, *p* = 0.002, η^2^ = 0.12). Additionally, intervention participants showed significantly lower scores on the Maslach Burnout Inventory (MBI) Emotional Exhaustion subscale compared with control participants (*F*(1, 73) = 5.68, *p* = 0.02, η^2^ = 0.04). Finally, intervention teachers reported marginally significantly lower scores on the Perceived Stress (PS) scale compared with the control participants, (F(1, 73) = 3.71, *p* = 0.06, η2 = 0.04).

The ANCOVA results and adjusted means are displayed in Table 2.

### 3.3. Results for the Trajectory of Change

For teacher well-being (TSW total), a statistically significant interaction was found between the intervention conditions and time points (*F*(2, 136) = 3.60, *p* = 0.03, η^2^ = 0.02). Post-hoc comparisons showed that participants in the control group demonstrated a significant worsening in well-being from baseline (M_adj_ = 3.27, SE = 0.06) to follow-up (M_adj_ = 3.08, SE = 0.07; *t*(37) = 2.83, *p* = 0.02), as well as from post-intervention (M_adj_ = 3.22, SE = 0.07) to follow-up (*t*(37) = 2.410, *p* = 0.05). In contrast, the intervention teachers maintained their well-being scores across all three time points (i.e., there were no significant differences between the pre-intervention (M_adj_ = 3.22, SE = 0.07), post-intervention (M_adj_ = 3.35, SE = 0.06), or follow-up (M_adj_ = 3.28, SE = 0.06) scores.

Scores on self-compassion (SCS-CZ scale) demonstrated an interaction of marginal significance between the intervention conditions and time points (*F*(2, 134) = 2.79, *p* = 0.07, η^2^ = 0.01). Surprisingly, participants in the control group demonstrated a significant increase in self-compassion scores from baseline (M_adj_ = 3.11, SE = 0.07) to follow-up (M_adj_ = 3.27, SE = 0.06; *t*(37) = −3.31, *p* = 0.01) and from post-intervention (M_adj_ = 3.10, SE = 0.06) to follow-up (*t*(37) = −4.031, *p* < 0.001). The intervention teachers maintained their self-compassion scores across all three time points (i.e., there were no significant differences between the pre-intervention (M_adj_ = 3.19, SE = 0.09), post-intervention (M_adj_ = 3.32, SE = 0.07), or follow-up (M_adj_ = 3.30, SE = 0.08) scores. The interaction between group and time was not statistically significant for any of the remaining outcome variables (i.e., USE, PS, PHQ, GAD, or MBI).

## 4. Discussion

The purpose of the present study was to evaluate the effect of a mindfulness and self-compassion-based program on primary school teachers in the Czech Republic with regard to their levels of well-being, self-efficacy, self-compassion, stress, depression, anxiety, and burnout. The findings from the primary analyses indicate that teachers who received the program showed beneficial post-test effects on several outcome variables as compared to teachers in the control group, confirming our initial hypotheses. Specifically, the intervention group teachers reported higher post-test self-efficacy and self-compassion, and lower depression, anxiety, and emotional exhaustion than participants in the control group. In addition, the primary analyses showed that the intervention-group teachers had a tendency toward higher post-test well-being and lower stress compared to the control group teachers.

Previous research [19] involving primary school teachers and mindfulness-based programs has shown similar outcomes. For example, a study of Croatian educators who received the CARE program similarly reported improved levels of self-compassion and maintained them at follow-up. In the same way, a recent study that used the MSC program [24] also found similar benefits. Findings from that study identified that being mindfully self-compassionate supported teachers’ self-awareness, enabled recognition of their common humanity, and supported the teachers to respond to challenging moments with self-kindness, which contributed to their well-being. These teachers also reported feeling more clear-headed and able to make better informed decisions, communicate more effectively with others, and persist with responding to challenging situations. In addition, offering themselves compassion as a way of regulating their emotional state appeared to enable the teachers to respond more compassionately to others [24]. This literature aligns with the results of the present study regarding well-being, self-compassion, and other variables connected with emotional regulation.

In addition, the current findings regarding teachers’ reduced levels of stress, depression, anxiety, and burnout are similar to those from previous research [3], such as a study that demonstrated that self-compassion practice helped to mitigate teachers’ empathy fatigue and burnout [26], which are both essential aspects of mental health for teachers. Recent research also indicates that self-compassion supports teachers in dealing with the stress of teaching [44] and demonstrates that the earlier the use of self-compassion, the more quickly emotional recovery occurred, suggesting that self-compassion is a helpful method of emotional regulation.

Examining the three time points across the school year indicated that the control teachers, who did not receive the intervention, showed a decline in their well-being. This is similar to the results of Harris and colleagues [2], who found that the control teachers showed increased burnout and increasing physiological distress over the course of the school year. In contrast, the intervention group maintained their well-being throughout the school year, without any significant change over time. This finding suggests a “protective effect” for the intervention group against a worsening well-being over the course of the school year. The study showed that providing teachers with emotion regulation, self-compassion, and mindfulness tools can lead to the maintenance of well-being throughout the school year. It would be helpful for future studies to examine these types of interventions for teachers with a larger sample size.

A counterintuitive finding emerged for the trajectory of change analysis; namely, that the control participants reported an increase in self-compassion from baseline to post-test and from baseline to follow-up, whereas the intervention participants showed no change across the three time points. This is a difficult finding to explain, but it is possible that the change in the control group reflected regression to the mean, as the control-group baseline score may have been a “floor effect” that increased over time. That said, it is important to note that this was a marginally significant finding and thus should be interpreted with caution and explored in future research.

In addition, further studies need to continue exploring how these programs might impact teachers’ physical health, their relationships with students, and the students’ outcomes.

## 5. Conclusions

In these challenging times, it is essential to continue addressing the issue of stress in the teaching profession. This study highlights the value of preventive interventions to support the mental and emotional health of primary school teachers in the Czech Republic.

This study had a number of limitations. First, as this study was a quasi-experimental design, the schools were not randomly assigned to the intervention and control groups. Non-random assignment carries several risks, such as potential bias resulting from self-selection into the intervention, as well as the introduction of confounding variables such as pre-existing differences in school culture, climate, etc. With this in mind, future studies should attempt to replicate the current findings using a fully randomized trial. In addition, there is a debate regarding whether to use ANCOVA or change scores when analyzing non-experimental longitudinal data [45]. The ANCOVA approach was deemed appropriate in the current study, as the baseline score was assumed to be the most relevant potential covariate; however, future research should carefully consider whether ANCOVA or change scores would be more appropriate. Finally, the study relied solely on teacher self-reports; thus, future studies should examine additional sources, including observational data, physical health, and/or student reports of the class climate and their relationships with teachers.

Future studies in the Czech Republic should also focus on mindful self-compassion for students, especially in light of the fact that there is already evidence in non-Czech populations suggesting that students who use well-being practices, such as gratitude or self-compassion, report increases in well-being and decreases in ill-being [31]. In the same regard, studies that have shown the benefits of self-compassion for teachers, in terms of an increasing self- efficacy and well-being, also show how those benefits can impact students’ perceptions of the quality of their teachers’ involvement in the classroom and their teachers’ social competencies [32].

In conclusion, this study was the first opportunity in the Czech Republic to examine teacher well-being and the effects of implementing a preventive mental-health-focused intervention in Czech schools. The use of self-compassion and mindfulness programs for teachers allows not only for strategies or tools to manage teacher stress, but also to improve other outcomes such as well-being, self-efficacy, self-compassion, depression, anxiety, or burnout. The results of this study have led to new funding for future studies related to mindfulness for primary school students and teachers in the Czech Republic. By prioritizing the mental and emotional health of primary school teachers, it is possible to continue to contribute to professional teacher development and to the education system in the Czech Republic.

## Figures and Tables

**Table 1:** Unadjusted pre-test (Time 1—T1), post-test (Time 2—T2), and follow up (Time 3—T3) means and standard deviations (in brackets) for all outcome variables for the control group and the intervention group.

Outcome Variable	Control Group			Intervention Group		
	T1	T2	T3	T1	T2	T3
TSW total (well-being)	3.29 (0.38)_n = 44_	3.24 (0.40)_n = 49_	3.08 (0.40)_n = 45_	3.18 (0.43)_n = 43_	3.35 (0.38)_n = 36_	3.29 (0.34)_n = 37_
TSW efficacy	3.12 (0.51)_n = 44_	3.12 (0.54)_n = 49_	2.99 (0.51)_n = 45_	3.02 (0.50)_n = 43_	3.19 (0.50)_n = 35_	3.25 (0.47)_n = 36_
TSW connectedness	3.45 (0.45)_n = 44_	3.36 (0.48)_n = 49_	3.17 (0.50)_n = 45_	3.33 (0.53)_n = 43_	3.48 (0.44)_n = 36_	3.32 (0.46)_n = 37_
USE (self-efficacy)	3.93 (0.43)_n = 44_	3.85 (0.43)_n = 49_	3.79 (0.47)_n = 45_	3.81 (0.48)_n = 43_	4.02 (0.41)_n = 36_	3.89 (0.36)_n = 37_
SCS (self-compassion)	3.08 (0.44)_n = 44_	3.13 (0.39)_n = 49_	3.25 (0.41)_n = 45_	3.20 (0.50)_n = 43_	3.33 (0.40)_n = 35_	3.31 (0.45)_n = 36_
PS (perceived stress)	1.09 (0.73)_n = 44_	1.39 (0.67)_n = 49_	1.16 (0.55)_n = 44_	1.09 (0.71)_n = 43_	1.13 (0.63)_n = 36_	1.17 (0.75)_n = 35_
PHQ (depression)	0.48 (0.48)_n = 44_	0.61 (0.41)_n = 49_	0.52 (0.41)_n = 44_	0.33 (0.35)_n = 43_	0.38 (0.31)_n = 36_	0.45 (0.41)_n = 35_
GAD (anxiety)	0.52 (0.55)_n = 44_	0.62 (0.45)_n = 49_	0.42 (0.42)_n = 44_	0.41 (0.39)_n = 43_	0.35 (0.31)_n = 36_	0.39 (0.51)_n = 35_
MBI emotional exhaustion (burnout)	2.13 (1.28)_n = 43_	2.21 (1.49)_n = 49_	2.15 (1.40)_n = 44_	1.94 (1.21)_n = 43_	1.49 (1.02)_n = 36_	1.38 (1.05)_n = 35_
MBI personal accomplishment (burnout)	4.76 (1.00)_n = 43_	4.57 (0.88)_n = 49_	4.43 (0.90)_n = 44_	4.58(0.93)_n = 43_	4.77 (0.81)_n = 36_	4.60 (1.17)_n = 35_

**Table 2:** Post-test scores and ANCOVA results for all outcome measures.

Outcome Variable	Intervention	Control	*F*	*p*
TSW total (well-being)	3.36 (0.05)	3.22 (0.05)	2.94	0.09
TSW efficacy	3.20 (0.07)	3.10 (0.07)	0.97	0.32
TSW connectedness	3.49 (0.06)	3.33 (0.06)	3.19	0.08
USE (self-efficacy)	4.03 (0.05)	3.86 (0.05)	5.36	0.02
SCS (self-compassion)	3.30 (0.05)	3.13 (0.04)	5.53	0.02
PS (perceived stress)	1.12 (0.10)	1.40 (0.09)	3.71	0.06
PHQ (depression)	0.40 (0.05)	0.59 (0.05)	6.01	0.02
GAD (anxiety)	0.35 (0.06)	0.64 (0.06)	10.42	0.002
MBI (burnout—emotional exhaustion subscale)	1.64 (0.16)	2.20 (0.15)	5.68	0.02
MBI (burnout—personal accomplishment subscale)	4.76 (0.11)	4.53 (0.10)	2.75	0.10

Note. Values are adjusted post-test means and standard errors (in brackets). Total *N* of participants, 104; intervention, *n* = 47; control, *n* = 54.

## Data Availability

The data presented in this study are available on request from the corresponding author.
